# [Retracted] Protection of telomeres 1 protein levels are associated with telomere length in gastric cancer

**DOI:** 10.3892/ijmm.2026.5896

**Published:** 2026-06-22

**Authors:** Kiyomu Fujii, Tomonori Sasahira, Yukiko Moriwaka, Naohide Oue, Wataru Yasui, Hiroki Kuniyasu

Int J Mol Med 21: 599-604, 2008; DOI: 10.3892/ijmm.21.5.599

Following the publication of the above article, a concerned reader drew the Editor's attention to the fact that the Pot1 data featured in Fig. 2A of the above paper were very similar to data for pJNK blots that appeared in Fig. 5 of an article in the journal *International Journal of Molecular Sciences* that featured two of the same authors, but was published more than 2 years earlier than the above article was received at *International Journal of Molecular Medicine*. Furthermore, in comparing these papers, it was subsequently noted by the Editor that, after horizontally flipping the bands, the data presented in the lanes that were adjacent to the pair of protein bands under consideration were different, suggesting that, in addition, this figure may also have been inappropriately edited.

After having conducted this internal investigation of the data in this paper, the Editor of *International Journal of Molecular Medicine* has decided that the article should be retracted from the publication on the grounds of an overall lack of confidence in the data. The authors were asked for an explanation to account for these concerns, but the Editorial Office did not receive a reply. The Editor sincerely apologizes to the readership for any incovenience caused, and we thank the reader for drawing this matter to our attention.

